# Structure and biosynthesis of carotenoids produced by a novel *Planococcus* sp. isolated from South Africa

**DOI:** 10.1186/s12934-022-01752-1

**Published:** 2022-03-19

**Authors:** Anesu Conrad Moyo, Laurent Dufossé, Daniele Giuffrida, Leonardo Joaquim van Zyl, Marla Trindade

**Affiliations:** 1 Institute for Microbial Biotechnology and Metagenomics (IMBM), Department of Biotechnology, University of the Western Cape, Bellville, Cape Town, 7535 South Africa; 2 BioCiTi Laboratory, 4th Floor Block B, Bandwidth Barn, Woodstock Exchange Building, 66-68 Albert Road, Woodstock, Cape Town, 7925 South Africa; 3 Chemistry and Biotechnology of Natural Products, CHEMBIOPRO, ESIROI Agroalimentaire, Université de La Réunion, 15 Avenue René Cassin, CS 92003, CEDEX 9, F-97744 Saint-Denis, France; 4 Università Degli Studi Di Messina, Dip. B.I.O.M.O.R.F, Polo Annunziata, 98168 Messina, ME Italy

**Keywords:** Halophilic bacteria, Pigments, Carotenoid biosynthesis, Whole genome sequencing

## Abstract

**Background:**

The genus *Planococcus* is comprised of halophilic bacteria generally reported for the production of carotenoid pigments and biosurfactants. In previous work, we showed that the culturing of the orange-pigmented *Planococcus* sp. CP5-4 isolate increased the evaporation rate of industrial wastewater brine effluent, which we attributed to the orange pigment. This demonstrated the potential application of this bacterium for industrial brine effluent management in evaporation ponds for inland desalination plants. Here we identified a C_30_-carotenoid biosynthetic gene cluster responsible for pigment biosynthesis in *Planococcus* sp. CP5-4 through isolation of mutants and genome sequencing. We further compare the core genes of the carotenoid biosynthetic gene clusters identified from different *Planococcus* species’ genomes which grouped into gene cluster families containing BGCs linked to different carotenoid product chemotypes. Lastly, LC–MS analysis of saponified and unsaponified pigment extracts obtained from cultures of *Planococcus* sp. CP5-4, revealed the structure of the main (predominant) glucosylated C_30_-carotenoid fatty acid ester produced by *Planococcus* sp. CP5-4.

**Results:**

Genome sequence comparisons of isolated mutant strains of *Planococcus* sp. CP5-4 showed deletions of 146 Kb and 3 Kb for the non-pigmented and “yellow” mutants respectively. Eight candidate genes, likely responsible for C_30_-carotenoid biosynthesis, were identified on the wild-type genome region corresponding to the deleted segment in the non-pigmented mutant. Six of the eight candidate genes formed a biosynthetic gene cluster. A truncation of *crtP* was responsible for the “yellow” mutant phenotype. Genome annotation revealed that the genes encoded 4,4′-diapolycopene oxygenase (CrtNb), 4,4′- diapolycopen-4-al dehydrogenase (CrtNc), 4,4′-diapophytoene desaturase (CrtN), 4,4′- diaponeurosporene oxygenase (CrtP), glycerol acyltransferase (Agpat), family 2 glucosyl transferase 2 (Gtf2), phytoene/squalene synthase (CrtM), and cytochrome P450 hydroxylase enzymes. Carotenoid analysis showed that a glucosylated C_30_-carotenoid fatty acid ester, methyl 5-(6-C_17:3_)-glucosyl-5, 6′-dihydro-apo-4, 4′-lycopenoate was the main carotenoid compound produced by *Planococcus* sp. CP5-4.

**Conclusion:**

We identified and characterized the carotenoid biosynthetic gene cluster and the C_30_-carotenoid compound produced by *Planococcus* sp. CP5-4. Mass-spectrometry guided analysis of the saponified and unsaponified pigment extracts showed that methyl 5-glucosyl-5, 6-dihydro-apo-4, 4′-lycopenoate esterified to heptadecatrienoic acid (C_17:3_). Furthermore, through phylogenetic analysis of the core carotenoid BGCs of *Planococcus* species we show that various C_30_-carotenoid product chemotypes, apart from methyl 5-glucosyl-5, 6-dihydro-apo-4, 4′-lycopenoate and 5-glucosyl-4, 4-diaponeurosporen-4′-ol-4-oic acid, may be produced that could offer opportunities for a variety of applications.

**Supplementary Information:**

The online version contains supplementary material available at 10.1186/s12934-022-01752-1.

## Background

Of the pigments produced by bacteria, carotenoids have diverse biological functions that include, coloration, photoprotection, light harvesting, and regulating the fluidity of the bacterial phospholipid bilayer membrane [1–6]. Biotechnologically, carotenoids have been utilized as food colorants, antioxidants, animal feed supplements, nutraceuticals, cosmetics, and pharmaceuticals [7–10].

Numerous bacterial species, including some from the *Planococcus* genus, are known to produce carotenoids [11–18]. The genus *Planococcus* comprises of; halophilic, aerobic, Gram-positive, motile cocci from various environments including saltern ponds [19–21]. The growth of *Planococcus* bacteria and other pigmented halophilic microorganisms in saltern ponds contributes to the red–orange coloration and enhanced evaporation rates of the brine during salt production [22–24]. Recently, we showed that culturing the orange pigmented *Planococcus* sp. CP5-4 isolate in industrial wastewater reverse osmosis brine effluent, at different salt compositions to those of saltern pond brines, resulted in a 20% increase in the evaporation rate of the brine. The resulting increase in the brine evaporation rate was attributed to the production of a carotenoid pigment by *Planococcus* sp. CP5-4, thus, showcasing the potential of pigmented halophilic bacteria as an environmentally friendly and sustainable alternative option to the use of chemical dyes for this purpose [25–29].

Although several strains belonging to the *Planococcus* genus have been reported as carotenoid producers, information on the identification and characterization of the genes and/or biosynthetic gene clusters (BGCs) responsible for carotenoid production in this genus is not extensively reported in the literature. As of the time of writing, the carotenoid BGCs of two strains, *Planococcus maritimus* iso-3 [30] and *Planococcus faecalis* AJ003^T^ [16, 31], have been analyzed, while only mentions of the existence of carotenoid biosynthetic genes in the sequenced genomes of *Planococcus maritimus* MKU009 [12], *Planococcus* sp. ANT_H30 [1], *Planococcus donghaensis* JH1T [32], *Planococcus halotolerans* SCU63^T^ [33], and *Planococcus rifietoensis* M8^T^ [34] has been made.

Furthermore, glucosylated carotenoid acid esters, in which a glucose molecule is esterified to both a triterpenoid carotenoid carboxylic acid and fatty acid (FA) [35], have been reported to be the end products of carotenoid biosynthesis in heterotrophic bacteria that include, but are not limited to, *Staphylococcus aureus* [36–38], *Planococcus maritimus* DSM 17275 [39, 40], *Planococcus faecalis* AJ003^T^ [16, 41], *Methylomonas* sp. [42], *Halobacillus halophilus* [43], *Bacillus indicus* [44, 45], and *Bacillus firmus* [45, 46]. However, the occurrence of glucosylated carotenoid esters in bacteria is often overlooked because of the routine use of saponification to hydrolyze the esters and remove the fatty acids before subsequent characterization of the carotenoid compounds. This practice results in the generation of data for glucosylated carotenoid compounds devoid of the esterified fatty acid chain [47–49] and the reporting of incomplete carotenoid structures in the literature.

Given the potential of carotenoids in industry and our demonstration that these may assist in improved evaporation rates of brines, we investigated the nature of the *Planococcus* sp. CP5-4 carotenoid biosynthetic pathway and the compounds produced. Here we present the genome sequence of *Planococcus* sp. CP5-4 and demonstrate that the proposed carotenoid biosynthetic pathway is indeed responsible for production of a glucosylated carotenoid ester.

## Results and discussion

### Isolation of carotenoid mutant strains, genome sequencing and classification

**Fig. 1:**
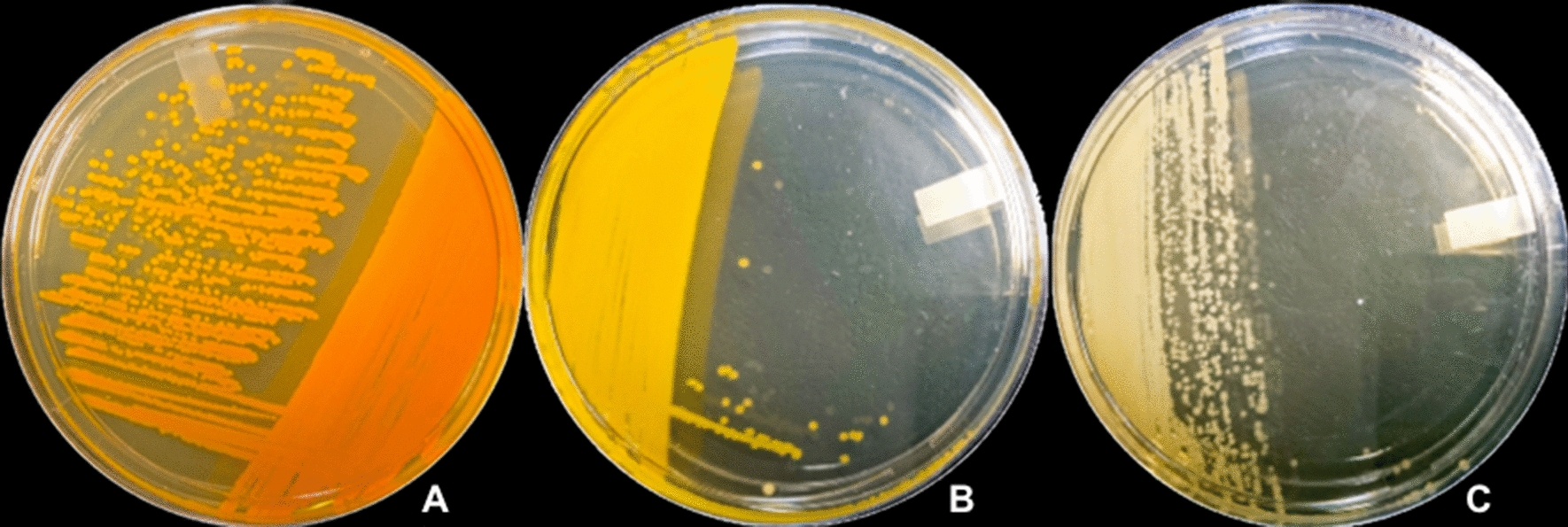
Wild type *Planococcus* sp. CP5-4 producing orange pigment (**A**), yellow (**B**), and unpigmented (**C**) mutant strains

Yellow and unpigmented mutant phenotypes designated *Planococcus* sp. CP5-4_YE and CP5-4_UN, respectively, were generated following MMS mediated mutagenesis (Fig. 1). Reports in the literature on the elucidation of pigment biosynthesis pathways in halophilic bacteria provide support for the production of white (or unpigmented) and yellow mutant strains following chemical mutagenesis using DNA alkylating agents [41, 48].

Genome sequencing of the *Planococcus* sp. CP5-4 strains and the analysis of the genomes were conducted to obtain an accurate classification of the wild type *Planococcus* sp. CP5-4 isolate that was previously tentatively identified as a novel species and to identify the pigment biosynthetic gene(s) or gene cluster(s) responsible for the expression of the orange pigment produced by this strain. The draft genome assembly of *Planococcus* sp. CP5-4 consists of 34 contigs that generated a genome size of 3 488 448 bp with a 47.5% G + C content (Additional file 1: Table S1 and S2). Of the 3612 predicted genes, 3 469 are protein-coding genes and 76 are RNAs (17 rRNA and 65 tRNA genes) (Additional file 1: Table S2). The wild-type *Planococcus* sp. CP5-4 genome grouped together with *Planococcus plakortidis*, *Planococcus maitriensis*, *Planococcus* sp. 002833405, *Planococcus rifietoensis, Planococcus maritimus*_B and *Planococcus maritimus* following classification (Additional file 1: Fig. S1). The placement of the *Planococcus* sp. CP5-4 strain in the tree suggests that *Planococcus* sp. CP5-4 is a new species in the *Planococcus* genus. However, when compared to genomes of related *Planococcus* species, the draft genome assembly for *Planococcus* sp. CP5-4 is unremarkable with respect to size, number of genes encoded, number of tRNA’s and G + C content (Additional file 1: Table S5).

#### Identification of the carotenoid biosynthetic gene cluster

**Fig. 2:**
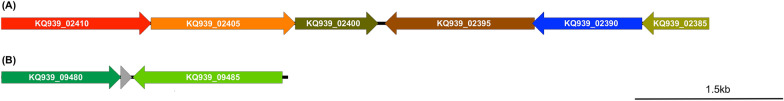
Organization of the ORFs predicted to be involved in carotenoid biosynthesis on **A** contig 1 and **B** contig 5 of the de novo assembled *Planococcus* sp. CP5-4 draft genome

Eight ORFs were predicted to be involved in carotenoid biosynthesis in the wild type *Planococcus* sp. CP5-4 genome. Six of the eight predicted ORFs were located on contig 1 as a 7876 bp biosynthetic cluster, while the remainder of the ORFs were located on contig 5 (Fig. 2).

A query of the protein sequence of the putative ORFs against the UniProtKB/Swiss-Prot database revealed that the sequences were related to those responsible for the biosynthesis of C_30_-carotenoids (Additional file 1: Table S3). Although functional similarity is challenging to infer when global sequence similarity is low, very different sequences can have largely similar activities based on conserved active-sites or functionally relevant regions [52]. As such, many proteins of barely detectable sequence similarity have the same function. It is generally considered that sequences with greater than 30–40% identity are functionally similar [53, 54]. Therefore, we predicted that the pigment produced by *Planococcus* sp. CP5-4 would be a C_30_-carotenoid. The CrtNb protein, a putative oxygenase, shared the lowest identity at 35.8%, suggesting that it might display different substrate specificity or kinetics compared to those that have been characterized [38].

Mapping the sequence reads from the *Planococcus* sp. CP5-4_YE and CP5-4_UN mutant strains to the wild type de novo assembled contigs revealed that copy number variants, specifically deletions measuring greater than 1 Kb in size from the wild type *Planococcus* sp. CP5-4 genome, were responsible for the altered phenotypes. For *Planococcus* sp. CP5-4_YE a 3 Kb sequence deletion resulted in the truncation of the *crtP* and three additional ORFs (Additional file 1: Figure S2). For *Planococcus* sp. CP5-4_UN a 146.691 Kb sequence deletion that included the six ORFs making up the carotenoid biosynthetic cluster resulted in the production of the unpigmented mutant strain following MMS mutagenesis (Additional file 1: Figure S3).

#### Carotenoid BGC core gene phylogenetic analysis

According to Takemura et al. [30], the genomes of several *Planococcus* species, such as *P*. *maritimus* iso-3, *P. faecalis* AJ003^T^, *P. plakortidis* and *P. halocryophilus* contain a similar carotenoid biosynthesis gene cluster that includes an additional biosynthesis gene to the six predicted on contig 1 of the wild type *Planococcus* sp. CP5-4 genome*.*

Guided by this information and the results from the functional annotation and identification of the carotenoid biosynthetic gene cluster in *Planococcus* sp. CP5-4, an investigation of the carotenoid BGCs from different *Planococcus* species was conducted to resolve their evolutionary relationship and group them into gene cluster families (GCFs) containing BGCs linked to a highly similar carotenoid product chemotype. The genetic diversity of BGCs within GCFs is often directly related to structural differences between their molecular products [55, 56]. Hence, the evolutionary relationships between the core genes of the carotenoid BGCs of the *Planococcus* species were inferred using CORASON.

**Fig. 3:**
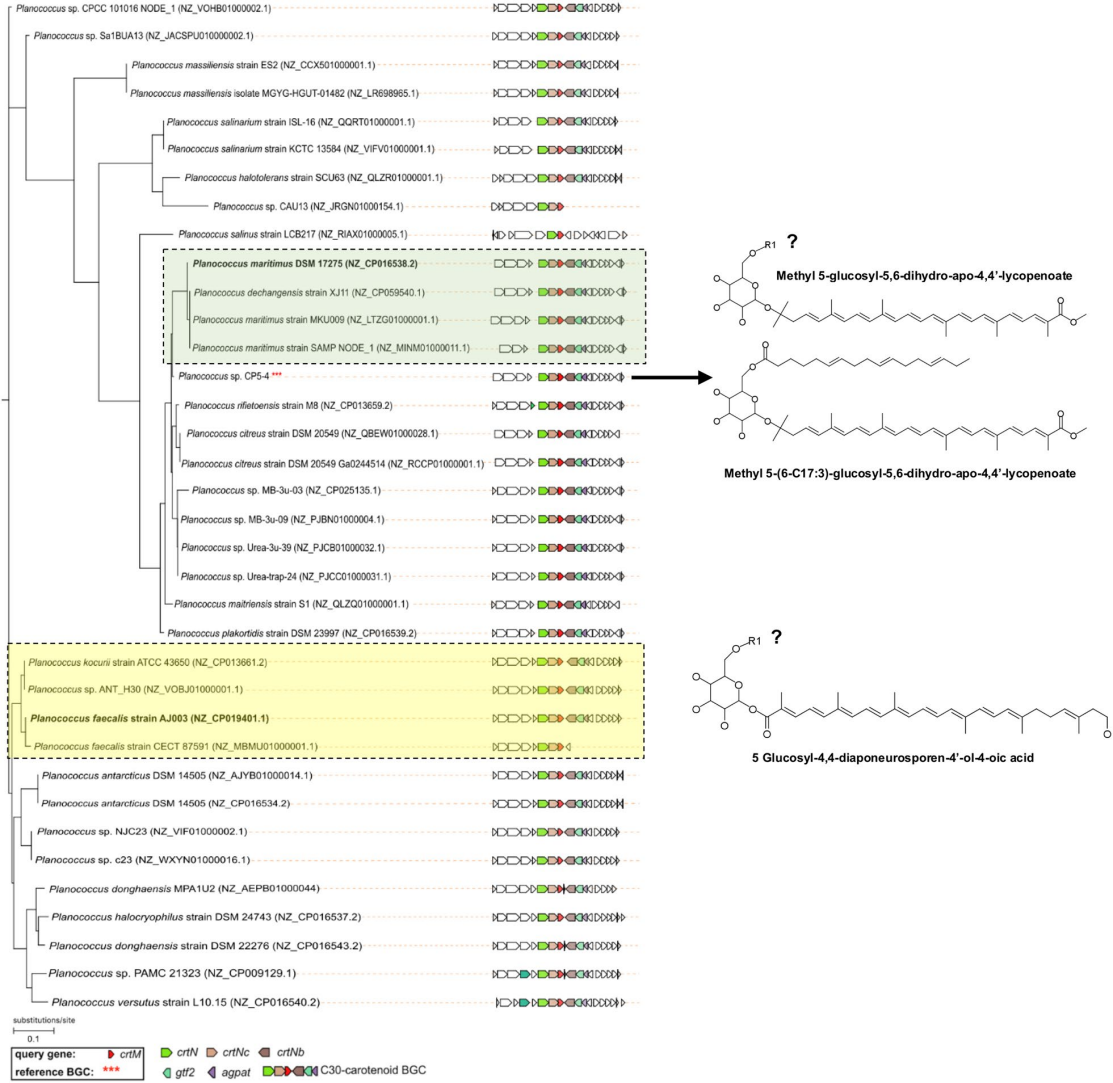
Phylogenetic tree of the carotenoid BGCs from various *Planococcus* bacterial genomes. Highlighted sections on the tree correspond to clades containing *Planococcus* bacterial species with characterized C_30_-carotenoid structures available in the literature

Using the *crtM* gene sequence as a query, 36 of 39 carotenoid BGCs detected by antiSMASH 5.0 in the queried *Planococcus* bacterial genomes were placed in the BGC phylogeny shown in Fig. 3. The 36 carotenoid BGCs were primarily grouped into two distinct clades. The first clade was named the ‘methyl 5-glucosyl-5, 6-dihydro-apo-4, 4′-lycopenoate clade′ because it contained *Planococcus maritimus* DSM 17275 which is a known methyl 5-glucosyl-5, 6-dihydro-apo-4, 4′-lycopenoate producer [13, 40]. The second clade of interest, termed the ‘5-glucosyl-4, 4-diaponeurosporen-4′-ol-4-oic acid clade,’ comprised BGCs with genes related to 5-glucosyl-4, 4-diaponeurosporen-4′-ol-4-oic acid biosynthesis which incorporates the recently characterized *Planococcus faecalis* AJ003 carotenoid BGC. The *Planococcus* sp. CP5-4 query BGC was placed into the methyl 5-glucosyl-5, 6-dihydro-apo-4, 4′-lycopenoate clade, indicating that the *Planococcus* sp. CP5-4 BGC should mediate synthesis of a methyl 5-glucosyl-5, 6-dihydro-apo-4, 4′-lycopenoate-like compound. Although several *Planococcus* carotenoid pathways group within these two clades, their genomic distance to characterized BGCs indicate that these species may produce novel chemotypes and should be a focus for future investigation.

### Pigment characterization

Analysis of saponified and unsaponified pigment extracts from *Planococcus* sp. CP5-4 were conducted in an attempt to resolve the complete structure of the carotenoid compound produced since the process of saponification results in the removal of fatty acid moieties that form part of the carotenoid structure in addition to removing lipids that may interfere with chromatographic separation [57]. The main compounds identified from the saponified pigment extract are presented in Table 1.

**Table 1:** UPLC-DA-MS detected compounds in the saponified pigment extract from *Planococcus* sp. CP5-4

*Peak	t_R_ (mins)	Formula	λ_max_ (nm)	Measured Mass (m/z)	Calculated Mass (m/z)	Error (ppm)
1	1.74	ND	271.1	ND	ND	ND
2	2.10	ND	236.1		ND	
3	3.04	C_37_H_53_O_8_	447, 467.12, 492.12	625.3732	625.3737	− 1.3
4	3.46	C_26_H_37_O_6_	268.12, 277.12, 283.12	445.2589	445.2590	− 0.2
5	6.06	ND	230	ND	ND	ND
6	7.62	C_30_H_43_O_2_	451.12	435.3277	435.3263	1.4

*ND* not determined

**Fig. 4:**
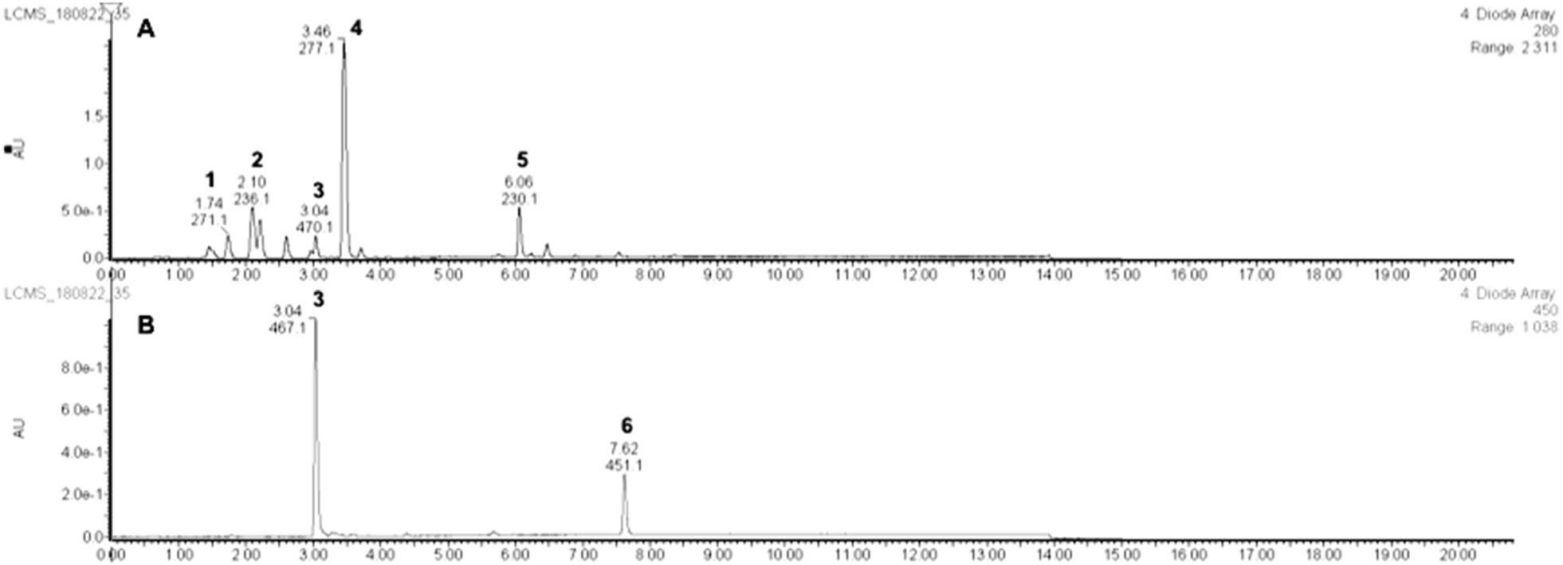
UPLC chromatograms of saponified pigment extract from *Planococcus* sp. CP5-4 recorded at **A** 280 nm and **B** 450 nm, respectively

*Peak numbers refer to Fig. 4

The pigment extracts were monitored at 280 nm and 450 nm to detect both the shorter polyene chain colourless carotenoids, which absorb maximally in the 280–320 nm region [58], and the long polyene chain coloured carotenoids that absorb in the visible region of the spectrum between 400 and 500 nm [59] Fig. 4).

**Fig. 5:**
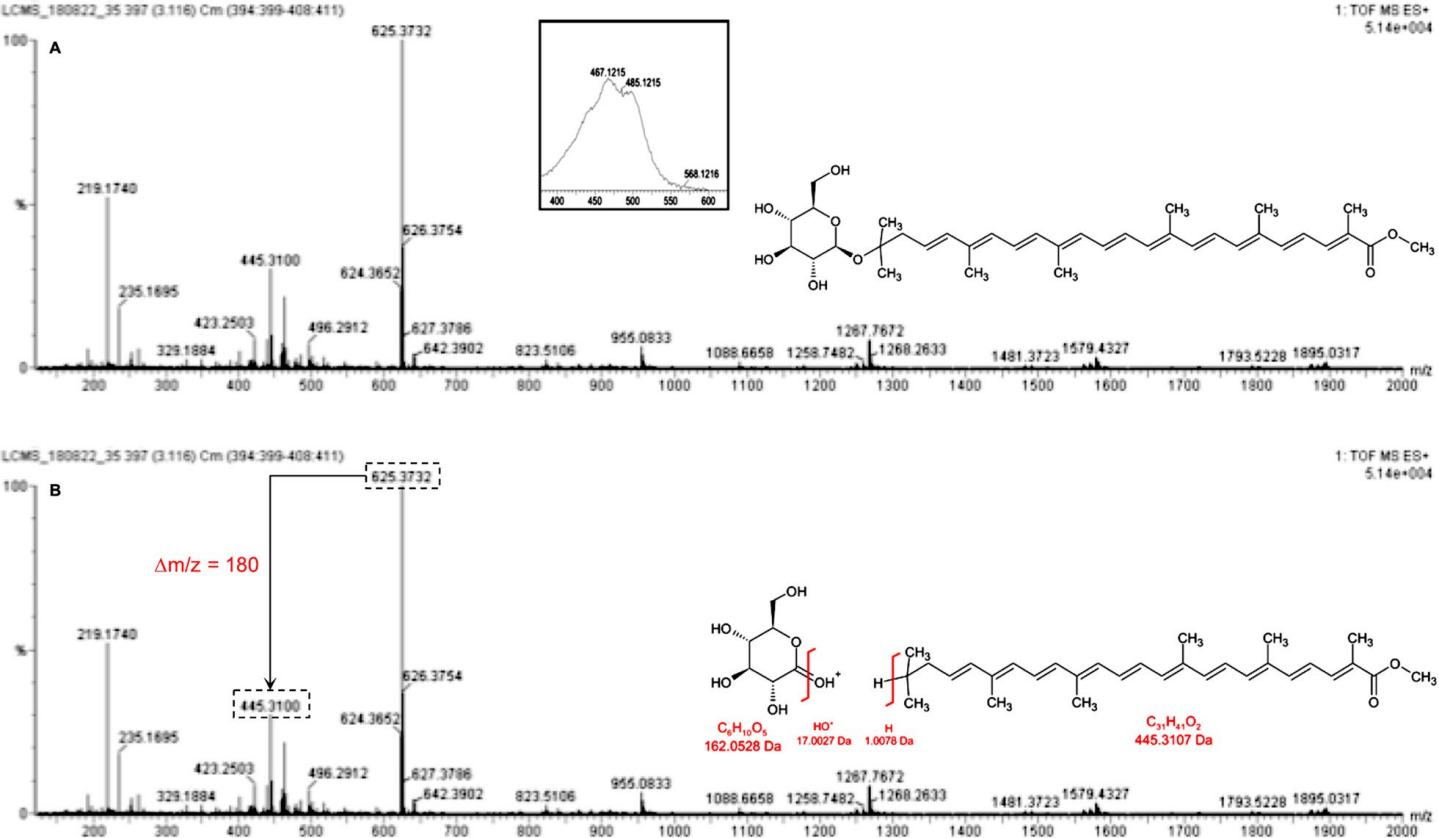
Characteristic MS and UV–Vis spectra of the main carotenoid compound detected in the saponified extract from *Planococcus* sp. CP5-4. **A** MS spectrum, UV–Vis spectrum, and proposed structure for the detected carotenoid compound; and **B** CID fragmentation pattern of methyl 5-glucosyl-5, 6-dihydro-apo-4, 4′-lycopenoate

The molecular formulae deduced for the compounds present in the saponified pigment extract from *Planococcus* sp. CP5-4 were mostly for C_30_ compounds (Table 1). Among these, the dominant peak (peak 3) in the chromatogram obtained at 450 nm in Fig. 4 B was taken to represent the most abundant C_30_-carotenoid compound in the saponified extract. The [M + H]^+^ peak of this compound had a *m*/*z* of 625.3732 (calcd: 625.3737; error:—1.3 ppm), and a predicted molecular formula of C_37_H_53_O_8._ The compound was therefore deduced to be methyl 5-glucosyl-5, 6-dihydro-apo-4, 4′-lycopenoate with a molecular formula of C_37_H_52_O_8_ informed by comparison with the MS data of the C_30_ carotenoids previously reported [32, 33] (Fig. 5A). The presence of a hexose sugar (*Δm/z* of 180 Fig. 5B) on the structure of methyl 5-glucosyl-5, 6-dihydro-apo-4, 4′-lycopenoate was revealed by the fragmentation peak with a measured *m/z* of 445.31 [M + H] ^+^ (error: -0.2 ppm). The 445.31 mass can be accounted for as the difference of 180 (loss of glucose) in the mass of methyl 5-glucosyl-5, 6-dihydro-apo-4, 4′-lycopenoate detected following CID (Figs. 5B).

**Fig. 6:**
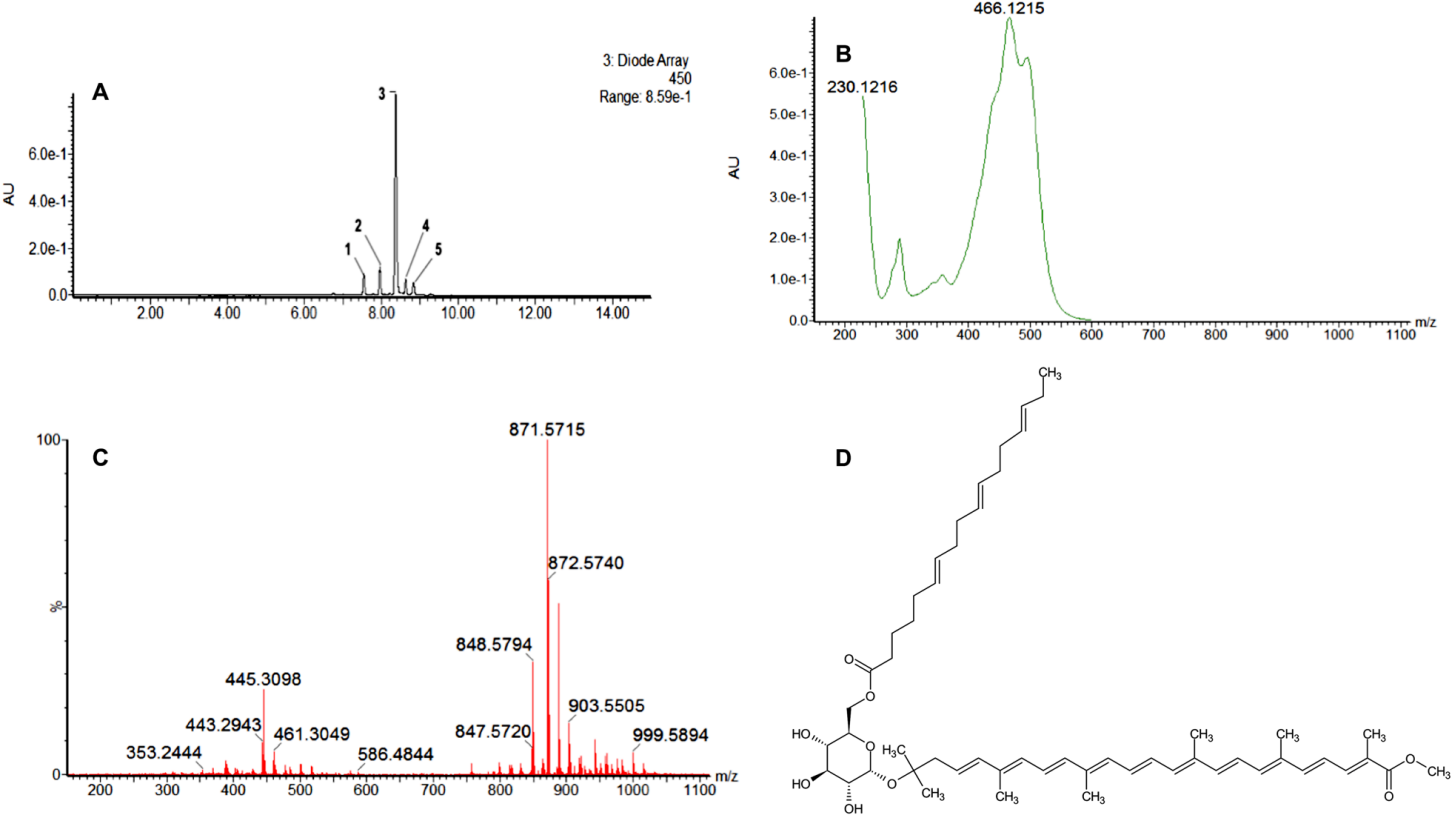
UPLC chromatogram for unsaponified pigment extract from *Planococcus* sp. CP5-4 (**A**), UV–Vis spectrum of the unsaponified pigment extract (**B**), MS-spectrum (**C**), and structure of the unsaponified methyl 5-(6-C_17:3_)-glucosyl-5, 6′-dihydro-apo-4, 4′-lycopenoate (**D**)

When analysing the unsaponified carotenoid extract, peak 3 eluting at 8 min 37 s was dominant with a UV–Vis spectral absorption maximum of 466 nm (Fig. 6A) and an absorption spectrum typical of a methyl-glucosyl-dihydro-apo-4, 4′-lycopenoate fatty acid ester [44, 60]. A base peak with *m/z* 871.5715 [M + H] ^+^ was obtained from the fragmentation of parent ion for peak 3 (Fig. 6C). The presence of an additional fatty acid moiety is revealed by subtracting the *m/z* of 445.3098 from the base peak of the unsaponified carotenoid (*m/z* 871.5715 [M + H] ^+^) to give a *Δm/z* of 426. Removing the mass of the glucosyl moiety (162) yields a remainder of* m/z* 264.1993, likely representing a C_17:3_ fatty acid moiety (Fig. 6D). Considering that a H_2_O molecule was eliminated during the esterification process the mass of the fatty acid moiety becomes 264.1993, which represents that of a C_17:3_—heptadecatrienoic acid. Thus, peak 3 in Fig. 6A was designated as methyl 5-(6-C_17:3_)-glucosyl-5, 6-dihydro-apo-4, 4’-lycopenoate derived from the parent mass of 871.5715 [M + H] ^+^ comprised of a hexose sugar (*Δm/z* 162) esterified to a heptadecatrienoic acid (*Δm/z* 264) and attached to 445.31 [M + H] ^+^ dihydro-methyl-apo-4, 4’-lycopenoate (Fig. 6C and D).

Although several polyunsaturated fatty acids have been reported to be produced by bacteria [61–64], prior to this study there has been no report of the production of an odd chain polyunsaturated fatty acid by species in the *Planococcus* genera.

The production of the heptadecatrienoic acid esterified to methyl 5-glucosyl-5, 6-dihydro-apo-4, 4′-lycopenoate may be attributed to the possible conversion of succinyl-CoA to propionyl-CoA by *Planococcus* sp. CP5-4. Three ORFs coding for the large and small subunits of a methylmalonyl-CoA mutase, and YgFD; a protein forming a complex with the methylmalonyl-CoA mutase subunits in the pathway for conversion of succinyl-CoA to propionyl-CoA were identified (Additional file 1: Table S5). The condensation of both propionyl-CoA and malonyl-CoA results in the formation of 3-oxovaleryl-ACP, which is the launching point for odd-chain FA synthesis. This five-carbon compound goes through elongation, where two carbons are added in each cycle leading to synthesis of odd-chain FAs [65, 66]. Moreover, the introduction of the three double bonds to the heptadecanoic FA chain to produce the heptadecatrienoic acid may have been mediated by a cell membrane bound fatty acid desaturase, a putative protein that could fulfill this role was also identified (Additional file 1: Table S5).

**Fig. 7:**
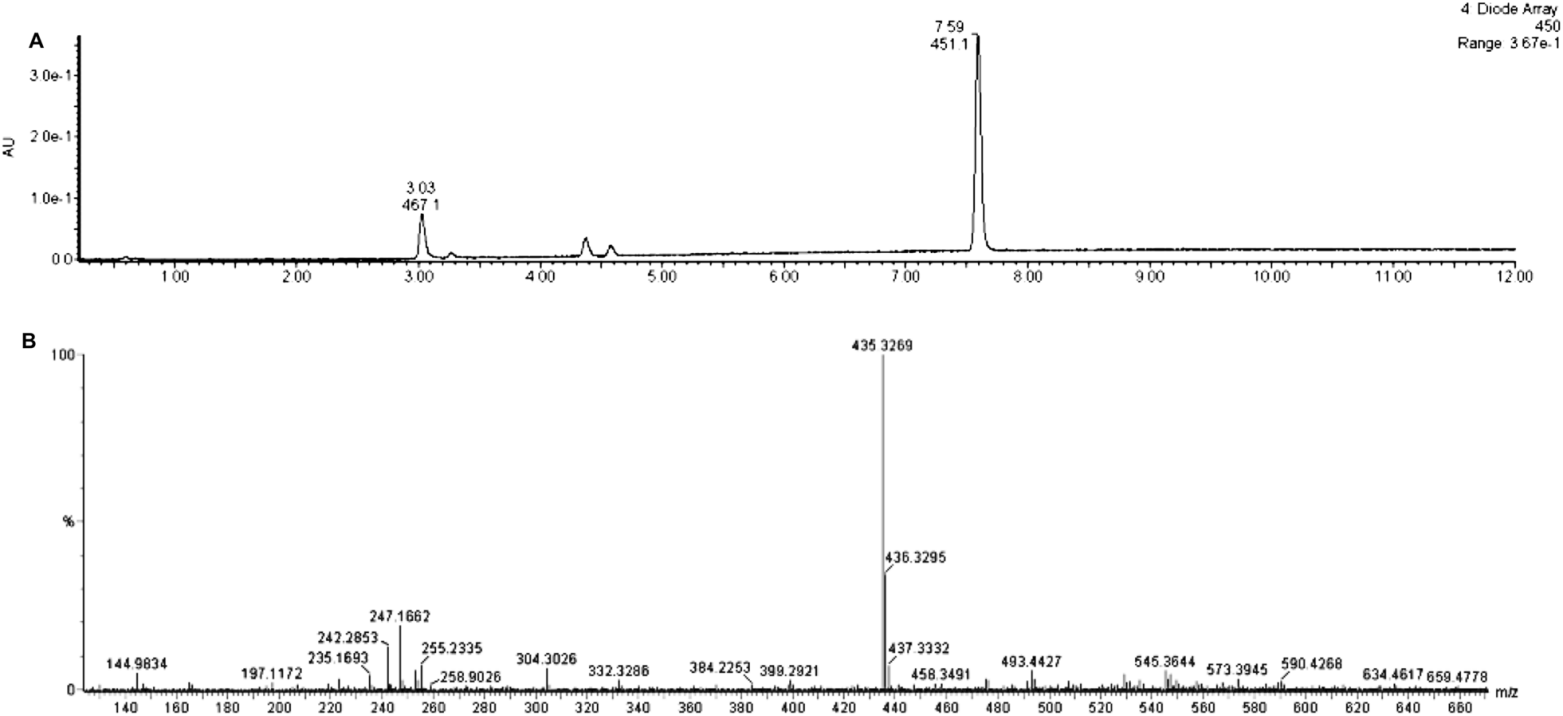
UPLC chromatogram for saponified pigment extract from yellow *Planococcus* sp. CP5-4 mutant recorded at 450 nm (**A**) and MS spectrum of the yellow extract (**B**)

Regarding the truncation of *crtP*, UPLC analysis of the saponified extract from the yellow mutant strain revealed peak 6, previously observed eluting as a minor peak in the chromatogram of the wild type strains’ saponified extract (Fig. 4A), as the predominant peak eluting at 7 min 59 s (Fig. 7A).

The aforementioned peak gave a visible spectral maximum at 451 nm and a [M + H] ^+^ ion peak at *m/z* 435. 3269 corresponding to a predicted formula of C_30_H_43_O_2_ (error: 1.4 ppm; Fig. 7B). These data suggest that the yellow pigment may be a putative glucosylated diapolycopene, supported by the yellow glucosylated diapolycopene and glucosylated diapolycopene fatty acid esters absorbing maximally between 449 to 454 nm from saponified and unsaponified carotenoid extracts from yellow *Bacillus* spore formers [67]. The truncation of *crtP* is theorized to have resulted in the translation of a nonfunctional enzyme that could not mediate the addition of a terminal O-methyl ester group to glucosylated diapolycopene during the biosynthesis of the orange methyl 5-(6-C17:3)-glucosyl-5, 6-dihydro-apo-4, 4′-lycopenoate, thus resulting in the production of the yellow pigment. According to Sy et al. [68], the main structural difference between the glucosylated orange and yellow C_30_ pigments is the absence of the terminal O-methyl ester group in the yellow pigment.

## Conclusions

Through isolation of pigment mutants combined with genome sequencing and mass-spectrometry we identified the genes responsible for the biosynthesis of a C_30_-carotenoid compound produced by *Planococcus* sp. CP5-4. The unsaponified pigment consists of methyl 5-glucosyl-5, 6-dihydro-apo-4, 4′-lycopenoate esterified to an odd chain unsaturated (C_17:3_) heptadecatrienoic acid. It is possible that the production of the unsaturated FA esterified to glucosyl-5, 6-dihydro-apo-4, 4′-lycopenoate is a mechanism for *Planococcus* sp. CP5-4 to maintain membrane fluidity. Furthermore, through genomic core phylogenetic analysis of the carotenoid BGCs of species within the *Planococcus* genus we hypothesize that carotenoid chemotypes apart from methyl 5-glucosyl-5, 6-dihydro-apo-4, 4′-lycopenoate and 5-glucosyl-4, 4-diaponeurosporen-4’-ol-4-oic acid may be produced thus paving the way for future studies to conduct an informed determination of the potential applications of these molecules.

## Methods

### Organism cultivation

The *Planococcus* sp. CP5-4 strain used in this study originated from a collection of bacterial cultures previously isolated from brine samples taken from the Cerebos crystallizer salt ponds in Velddrif, Western Cape, South Africa (S 32°47′10,632, E 18°10′9,499) [69]. *Planococcus* sp. CP5-4 was grown in complex medium (Tryptic Soy Broth; Sigma-Aldrich, Darmstadt, Germany, supplemented with 5% (w/v) NaCl and 0.5 M sorbitol). Where necessary 15 g/L of agar were added to the broth prior to sterilizing. The inoculated broth was incubated at room temperature with shaking at 150 rpm, while the agar plates were incubated at 28 °C.

### Random chemical mutagenesis

Random chemical mutagenesis was employed to generate pigment mutants to be used in the characterization of the pigment biosynthetic pathway. Mutagenesis was carried out on cells at the mid exponential phase of growth (OD660nm 0.7–0.75) as follows: The cells were harvested by centrifugation at 3214 ×*g* for 10 min, washed with, and re-suspended in 50 mL of the growth medium. The cell suspension was then concentrated 50-fold in quarter strength Ringer’s solution (Sigma-Aldrich, Steinheim, Germany) and methyl methanesulfonate (Sigma-Aldrich, Steinheim, Germany) added to a final concentration of 0.15 M. This mixture was inverted several times and incubated for 5 min at room temperature in a fume hood. At the end of the incubation period, the dosed wild type cells were washed thrice with 30 mL Ringer’s solution through repeated suspension and centrifugation for 10 min at 3 889 × *g* in a refrigerated centrifuge set to 20 °C. After the final washing step, the pelleted cells were re-suspended in 1 mL TSB containing 15%(v/v) glycerol and 5% (w/v) NaCl to make the mutant library, which was stored at − 80° C until needed. Screening of the library was conducted by spread plating a one in one thousand dilution of the library in Ringer’s solution on TSB-salt-sorbitol agar plates. Colonies were then visually inspected for pigmentation differing from that of the wild type after incubation at 28° C for 48 h.

### Genome sequencing and analysis

#### Sequencing, assembly and genome-based classification of Planococcus sp. CP5-4

Genomic DNA was extracted from cells pelleted from 5 mL cultures of the *Planococcus* sp. CP5-4 strains using a modified version of the method reported by Wang et al. [70]. The following modifications were made: lysozyme concentration was increased to 25 mg/mL, 0.2 mg/mL (final concentration) of proteinase K was added to the lysis buffer and cells were incubated overnight at 37 °C in the lysis buffer. The quality and quantity of the extracted DNA were verified through agarose gel electrophoresis and fluorometric measurement using a Qubit 2.0 fluorometer prior to library preparation using an Illumina Nextera XT kit following the manufacturer’s recommendations. The paired end libraries were sequenced on an in-house Illumina MiSeq instrument using the MiSeq v2 chemistry (Illumina, San Diego, CA, USA) yielding fastq files containing 2 × 250 bp reads. Following trimming of the reads, de novo assembly was performed using Geneious ver. 11.1.0 [71, 72] with default settings. Contigs with a minimum length less than 1000 bp were removed from the final assembly. The final numbers of contigs were 34, 43 and 45 for the wild type, yellow and unpigmented *Planococcus* CP5-4 strains, respectively. This Whole Genome Shotgun project was deposited in the DDBJ/ENA/GenBank database under Bio Project PRJNA738312 and Accession Numbers JAHREQ000000000.1, JAHPZO000000000.1 and JAHRBB000000000.1.

To obtain an accurate taxonomic classification of the *Planococcus* sp. CP5-4 strain, the contigs were queried using the Genome Taxonomy Database Toolkit (GTDB-Tk) [73] in the Kbase web portal [74, 75]. The GTDB-Tk was used in preference to the NCBI [76] database because the GTDB provides a comprehensive genome-based taxonomy with bacterial and archaeal taxa circumscribed on the basis of monophyly and relative evolutionary divergence [77] compared to the NCBI database, reported to show inconsistent taxonomies with many polyphyletic groupings for bacteria [73, 77, 78].

#### Annotation, and identification of carotenoid biosynthetic gene cluster

Functional annotation of the de novo assembled *Planococcus* sp. CP5-4 contigs was conducted in Blast2Go (B2G) ver. 5.2.2 [79, 80]. The annotations for the open reading frames (ORFs) predicted to be involved in carotenoid biosynthesis by B2G were validated by querying the translated gene sequences in the UniProtKB/Swiss-Prot database [81] for homologous sequences. The UniProtKB/Swiss-Prot database was used because it contains manually annotated records with information extracted from literature and curator-evaluated computational analysis that brings together experimental results, computed features and scientific conclusions [82, 83], as opposed to the computationally analyzed records awaiting full manual annotation obtained by the NCBI QBlast algorithm in the non-redundant nucleotide (nr/nt) collection database used by B2G. To confirm that the predicted ORFs were responsible for carotenogenesis, the paired-end sequence reads of the yellow and unpigmented mutant strains were mapped to the de novo assembled contig(s) of the *Planococcus* sp. CP5-4 wild type strain, which contained the predicted carotenogenesis ORFs using Geneious as the mapper. The sensitivity of the operation was set on ‘Medium-Sensitivity/Fast’ and the ‘Fine Tuning’ option set on ‘iterate’ up to five times to improve the results by aligning reads to each other in addition to the reference sequence. Following mapping, variant calling on the mapped data was conducted to identify the mutations responsible for the altered phenotypes of the mutant strains. Variants were detected in the mapped data using default settings in Geneious with the option to ‘Analyze the Effect of Polymorphisms on Translation’ chosen, and the default genetic code changed to ‘Bacterial’.

#### Carotenoid BGC genomic core phylogenetic analysis

The genomic core of carotenoid BGCs identified in 38 *Planococcus* genome sequences downloaded from the National Center for Biotechnology Information, U.S. National Library of Medicine database (https://www.ncbi.nlm.nih.gov/genome/browse#!/prokaryotes/planococcus) and that of *Planococcus* sp. CP5-4, were analyzed using CORASON software version 1.0 [56]. To conduct the analysis, the *Planococcus* genomes were first analyzed using antiSMASH 5.0 [84] at default settings and the predicted BGCs associated with the queried *Planococcus* genome sequences downloaded as.gbk files.

A database of all the identified carotenoid BGCs in the query sequences was created. The carotenoid BGC predicted to be present in the *Planococcus* sp. CP5-4 genome was chosen as the reference BGC for the database, while the *crtM* gene from the biosynthetic core of the reference BGC was chosen as the query protein. The choice for the reference BGC and query protein for the database was made after verification that: the reference BGC was one of the longest BGCs in the database, the query protein came from a biosynthetic core gene or an additional biosynthetic gene close to the core, and that the query protein was present in at least half of the BGCs in the database as suggested by Chanson, Moreau, and Duplais [85]. Once the reference BGC and protein query were selected for the database, the CORASON software (https://github.com/nselem/corason) was used to determine the genomic core of carotenoid BGCs, and to infer the evolutionary relationship of the BGCs.

### Pigment analysis

#### Carotenoid extraction

For carotenoid extraction, cells from a 5-day-old, 1 L culture were pelleted by centrifugation at 4 629 ×*g* at 4 °C for 15 min, and washed three times with sterile water. Cell pellets were dried and a mixture of acetone:methanol (7:3 v/v) containing 0.1% butylhydroxytoluene (BHT) as antioxidant added. The resulting cell suspensions were subjected to freeze—thaw cycles in liquid N_2_ to facilitate extraction of the pigment. The extracts were then centrifuged at 12,857 ×*g* for 10 min at 4 °C, and the coloured supernatant pipetted into foil-covered 50 mL Falcon tubes for protection from photo oxidation. Successive extractions were carried out on the cell pellets until both the solvent and the cells were colourless. The solvent phases were pooled together and evaporated to dryness under a N_2_ gas stream. Duplicate extractions were conducted for the wild type *Planococcus* sp. CP5-4 culture and one set of the extracts saponified concurrently with the yellow *Planococcus* sp. CP5-4 mutant extract as described by Cardinault et al. [86]. Pigment from the yellow *Planococcus* sp. CP5-4 mutant strain was extracted to identify the effects of the mutation(s) induced by MMS on the structure of the pigment.

Following saponification, the mixture was centrifuged (10 min, 4629 ×*g*, 4 °C) and the upper orange phase collected, washed with sterile water and recollected before being evaporated under N_2_ gas to produce an ‘oily’ red extract. The lower phase containing the saponified products of carotenoid esters was discarded, and the ‘oily’ red residue stored together with the dried unsaponified extract under nitrogen at − 80 °C before further processing. Extraction and saponification procedures were conducted in the dark.

#### Ultra-performance liquid chromatography (UPLC)

Prior to the determination of the composition of the pigment extracts, the unsaponified and saponified extracts were re-dissolved in 500 μL of acetone containing 0.1% BHT, loaded onto preparative thin layer chromatography (PLC) silica gel plates measuring 4 cm × 14 cm (60 F_254_ 2 mm; Merck, Darmstadt, Germany), which were developed in the dark in a chamber containing hexane: acetone (70:30) as mobile phase. The separated pigment phases were collected, dried under N_2_ stream, and the carotenoid compositions analyzed using a Waters Synapt G2 High-Definition (HD) quadrupole time-of-flight (qTOF) mass spectrometer (MS) connected to a Waters Acquity ultra-performance liquid chromatograph (UPLC) (Waters, Milford, MA, USA). For the analysis, 5 μL of the samples were injected into a Waters BEH C_18_, 2 × 100 mm, 1.7 μm column thermostated at 45 °C.

The column was initially eluted for 5 min using a gradient of 30% of solvent A (0.1% formic acid) and 62% acetonitrile containing 0.1% formic acid (solvent B), then for a further 25 min using 100% (solvent B). After which, the column was returned to the initial elution conditions (30% solvent A and 62% solvent B) and equilibrated over 30 min. In all instances the flow rate was maintained at 0.4 mL/min. The separation of components was monitored using a Waters eλ photodiode array detector set to measure in the 200–600 nm range and a mass spectrometer at the 150–1500 m/*z* range in MSe positive mode. Data was acquired using two separation functions; F1: at a low collision energy (4 V) and F2: using a collision energy ramp (25 − 60 V) to simultaneously acquire both unfragmented and fragmented data. Leucine enkephalin was used as lock mass (reference mass) for accurate mass determination and the instrument was calibrated weekly with sodium formate. The following MS settings were used: cone voltage of 15 V, desolvation temperature of 275 °C, desolvation gas at 650 L/h, and the rest of the MS settings optimized for best resolution and sensitivity. The identification of components was performed by the analysis of the absorbance spectra in connection with mass spectra using the Targertlynx module of the MassLynx ver. 4.1 software (Waters, Milford, USA).

## Supplementary Information

**Additional file 1: Table S1.** De novo assembly report for Planococcus sp. CP5-4 strains. **Table S2.** Features for the de novo assembled CP5-4 genome. **Table S3.** ORFs predicted to be associated with carotenoid biosynthesis on contigs 1 and 5 of the de novo assembled Planococcus sp. CP5-4 genome. **Table S4.** Average nucleotide identity comparison between CP5-4 and closely related GTDB Planococcus species. **Table S5.** Genome property comparison between CP5-4 and related Planococcus species. **Table S6.** Locus tags in CP5-4 involved in unsaturated fatty acid biosynthesis. **Figure S1.** Placement of the Planococcus CP5-4 strain in the GTDB bac 120 tree relative to other Planococcus species. The genome identifiers of the organisms are shown on the tree branches, where appropriate, both the GTDB (release.95) and unfiltered NCBI taxonomy names are given in parenthesis. The tree is based on the topology of the genomes and not on bootstrap resampling as this is computationally prohibitive and consequently may over-classify genomes relative to manual curation based on unsupported affiliations of user genomes to reference taxa. Part of the GTDB bac120 genome tree is shown. **Figure S2.** Deleted 3 Kb sequence region from the wild type CP5-4 strain's genome to produce a truncation of crtP and expression of the yellow mutant phenotype. The black coloured ORF represents crtP while the red ORF represents the flavodoxin reductase gene. The respective locus tags for the ORFs are also shown. Figure not drawn to scale. **Figure S3.** Deleted 146.691 Kb sequence region from the wild type CP5-4 strain's genome to produce the unpigmented mutant phenotype. The red ORFs represent the predicted carotenoid biosynthetic gene cluster while the green ORFs represent the additional genes that were also deleted as a result of DNA repair following MMS mutagenesis. The position of two insertion sequences ISBsp5 and ISBce1 (Table S4) that may have mediated the deletion are shown. Figure not drawn to scale.
