# Supplementary material for: Structure and biosynthesis of carotenoids produced by a novel Planococcus sp. isolated from South Africa
Source: Microb Cell Fact. 2022 Mar 19;21:43. doi: 10.1186/s12934-022-01752-1 (PMC8933910; doi:10.1186/s12934-022-01752-1)
Supplement: Supplementary file 1 — Additional file 1: Table S1. De novo assembly report for Planococcus sp. CP5-4 strains. Table S2. Features for the de novo assembled CP5-4 genome. Table S3. ORFs predicted to be associated with carotenoid biosynthesis on contigs 1 and 5 of the de novo assembled Planococcus sp. CP5-4 genome. Table S4. Average nucleotide identity comparison between CP5-4 and closely related GTDB Planococcus species. Table S5. Genome property comparison between CP5-4 and related Planococcus species. Table S6. Locus tags in CP5-4 involved in unsaturated fatty acid biosynthesis. Figure S1. Placement of the Planococcus CP5-4 strain in the GTDB bac 120 tree relative to other Planococcus species. The genome identifiers of the organisms are shown on the tree branches, where appropriate, both the GTDB (release.95) and unfiltered NCBI taxonomy names are given in parenthesis. The tree is based on the topology of the genomes and not on bootstrap resampling as this is computationally prohibitive and consequently may over-classify genomes relative to manual curation based on unsupported affiliations of user genomes to reference taxa. Part of the GTDB bac120 genome tree is shown. Figure S2. Deleted 3 Kb sequence region from the wild type CP5-4 strain's genome to produce a truncation of crtP and expression of the yellow mutant phenotype. The black coloured ORF represents crtP while the red ORF represents the flavodoxin reductase gene. The respective locus tags for the ORFs are also shown. Figure not drawn to scale. Figure S3. Deleted 146.691 Kb sequence region from the wild type CP5-4 strain's genome to produce the unpigmented mutant phenotype. The red ORFs represent the predicted carotenoid biosynthetic gene cluster while the green ORFs represent the additional genes that were also deleted as a result of DNA repair following MMS mutagenesis. The position of two insertion sequences ISBsp5 and ISBce1 (Table S4) that may have mediated the deletion are shown. Figure not drawn to scale. [file 12934_2022_1752_MOESM1_ESM.docx]

**Supplementary information: Structure and biosynthesis of carotenoids produced by a *Planococcus* sp. isolated from South Africa.**

Anesu Conrad Moyo^a^, Laurent Dufosse^b^, Daniele Giuffrida^c^, Leonardo Joaquim van Zyl^a^, and Marla Trindade^a,^ *

^a^ Institute of Microbial Biotechnology and Metagenomics (IMBM), Department of Biotechnology, University of the Western Cape, 7535, Bellville, Cape Town, South Africa,

^b^ Chemistry and Biotechnology of Natural Products, CHEMBIOPRO, ESIROI Agroalimentaire, Université de La Réunion, 15 Avenue René Cassin, CS 92003, CEDEX 9, F-97744 Saint-Denis, France.

^c^ Università degli Studi di Messina, Dip. B.I.O.M.O.R.F., Polo Annunziata, 98168 Messina (ME) - Italy.

# *De novo* assembly statistics for CP5-4 strains

Table S1 *De novo* assembly report for *Planococcus* sp. CP5-4 strains

| CP5-4 strain | Number of reads | Number of Contigs | Min length (bp) | Max length (bp) | N50 | L50 | Coverage |
| --- | --- | --- | --- | --- | --- | --- | --- |
| WT | 2 308 726 | 34 | 1 014 | 574 799 | 241 991 | 4 | 103.1 x |
| YE | 2 509 100 | 43 | 1 115 | 462 557 | 240,195 | 5 | 112 x |
| UN | 2 223 122 | 45 | 1 028 | 564 982 | 294,935 | 4 | 99.2 x |

Table S2 Features for the *de novo* assembled CP5-4 genome

| **Attributes** | **Value** |
| --- | --- |
| Genome size* | 3 488 448 |
| G+C content (%) | 47.5 |
| Number of ORFs | 28 863 |
| Number of genes | 3 557 |
| Coding Sequences (CDSs; total) | 3 454 |
| Protein coding genes | 3 415 |
| tRNA | 59 |
| rRNA | 17 |
| Genes per strand (+/-) | 1 790 /  1 679 |
| Length of genes (min/max) * | 114 /  4 590 |
| Average gene length* | 844.73 |

*Length in base pairs

Table S3 ORFs predicted to be associated with carotenoid biosynthesis on contigs 1 and 5 of the de novo assembled *Planococcus* sp. CP5-4 genome

| ORF locus tag | Size (bp) | B2G Annotation | UniprotKB/Swiss-Prot closest homolog | % Identity | Assigned Function |
| --- | --- | --- | --- | --- | --- |
| KQ939_02385 | 678 | Glycerol acyltransferase (*agpat*) | Acyl-phosphate glycerol 3-phosphate acyltransferase Protein (A0A2I0ESI3; *Planococcus sp*. Urea-3u-39) **^a^** | 94.6 | Mediates the transfer of an acyl group from one compound (donor) to another (acceptor). |
| KQ939_02390 | 1104 | Glucosyl transferase, family 2 (*gtf2*) | Glycosyl transferase family 2 protein (A0A0U2PCT2; *Planococcus rifietoensis*) **^a^**. | 94.6 | Mediates the transfer of a glycosyl group from a donor compound to an acceptor. |
| KQ939_02395 | 1506 | Apo-4, 4’-lycopene oxygenase (*crtNb)* | 4,4'-diapolycopene oxygenase (Q4VKU9*; Methylomonas* sp.) **^b^**. | 35.8 | Mediates the oxidation of the terminal methyl side groups of 4,4'-diapolycopene to yield 4,4'-diapolycopen-4,4'-dial via the aldehyde intermediate 4,4'-diapolycopen-al. Also, able to catalyze the oxidation of the terminal methyl side group of 4,4'-diaponeurosporene to form 4,4'-diaponeurosporen-4-al during C_30_ carotenoid biosynthesis. |
| KQ939_02405 | 1452 | Apo-4, 4’-lycopene aldehyde oxidase (*crtNc*) | 4,4'-diapolycopen-4-al dehydrogenase or alternatively 4,4'-diapolycopen-4-al oxidase (P0DPE9 (CRTNC_BACID); *Bacillus indicus*) **^b.^** | 53.5 | Involved in the biosynthesis of C_30_ carotenoids. Mediates the oxidation of 4,4'-diapolycopen-4-al to yield 4,4'-diapolycopen-4-oic acid. |
| KQ939_02400 | 831 | Phytoene synthase (*crtM*) | Phytoene/squalene synthase family protein (A0A3M8P827; *Planococcus salinus*) **^a.^** | 77.9 | Mediates the two steps reaction converting geranyl-geranyl diphosphate to phytoene *via* prephytoene diphosphate, or conjugation of two prenylated farnesyl pyrophosphate (FPP) precursor molecules to form 4,4’-diapophytoene or apo-4,4’-phytoene during C_50_, C_40_ or C_30_ carotenoid biosynthesis, respectively. |
| KQ939_02410 | 1527 | Apo-4, 4’-phytoene desaturase (*crtN*) | 4,4'-diapophytoene desaturase (Q4VKV1 (CRTN_METSP); *Methylomonas* sp.) **^b.^** | 52.3 | Involved in the biosynthesis of C_30_ carotenoids. Catalyzes four successive dehydrogenation reactions that lead to the introduction of four double bonds into 4,4'-diapophytoene (dehydrosqualene) to yield 4,4'-diapolycopene. |
| KQ939_09480 | 1200 | Cytochrome P450 hydroxylase | Cytochrome P450 107B1 (A0A098EMI8_9BACL; *Planococcus massiliensi*) **^a.^** | 77.3 | Mediates the hydroxylation of carotenoids. |
| KQ939_09485 | 1503 | CrtNb-like apo-4, 4’- terminal methyl oxidase (*crtP*) | 4,4'-diaponeurosporene oxygenase (Q4L978 (CRTP_STAHJ); *Staphylococcus haemolyticus* (strain JCSC1435)) **^b.^** | 60.3 | Involved in the biosynthesis of the yellow-orange carotenoid staphyloxanthin. Mediates the oxidation of the terminal methyl side group of 4,4'-diaponeurosporene to form 4,4'-diaponeurosporen-4-al. |

**^a.^** Unreviewed TrEMBLE top hit

**^b.^** Reviewed Swiss-Prot top hit

# Taxonomic classification of *Planococcus* sp. CP5-4

*Planococcus* sp. CP5-4 was grouped together with *Planococcus plakortidis*, *Planococcus maitriensis*, *Planococcus* sp. 002833405, *Planococcus rifietoensis,* *Planococcus maritimus*_B, and *Planococcus maritimus* following classification. The placement of the *Planococcus* sp. CP5-4 strain in the concatenated bacterial protein genome tree suggests that CP5-4 is a new species in the *Planococcus* genus. Designation of CP5-4 as a new species in the genus *Planococcus* is based on the assigned ANI of 89.49% to the closest related reference genome of *Planococcus* sp. 002833405 being less than the species-specific ANI circumscription radius of 95 % and the alignment fraction (AF) being greater than 0.65 (Table S4).


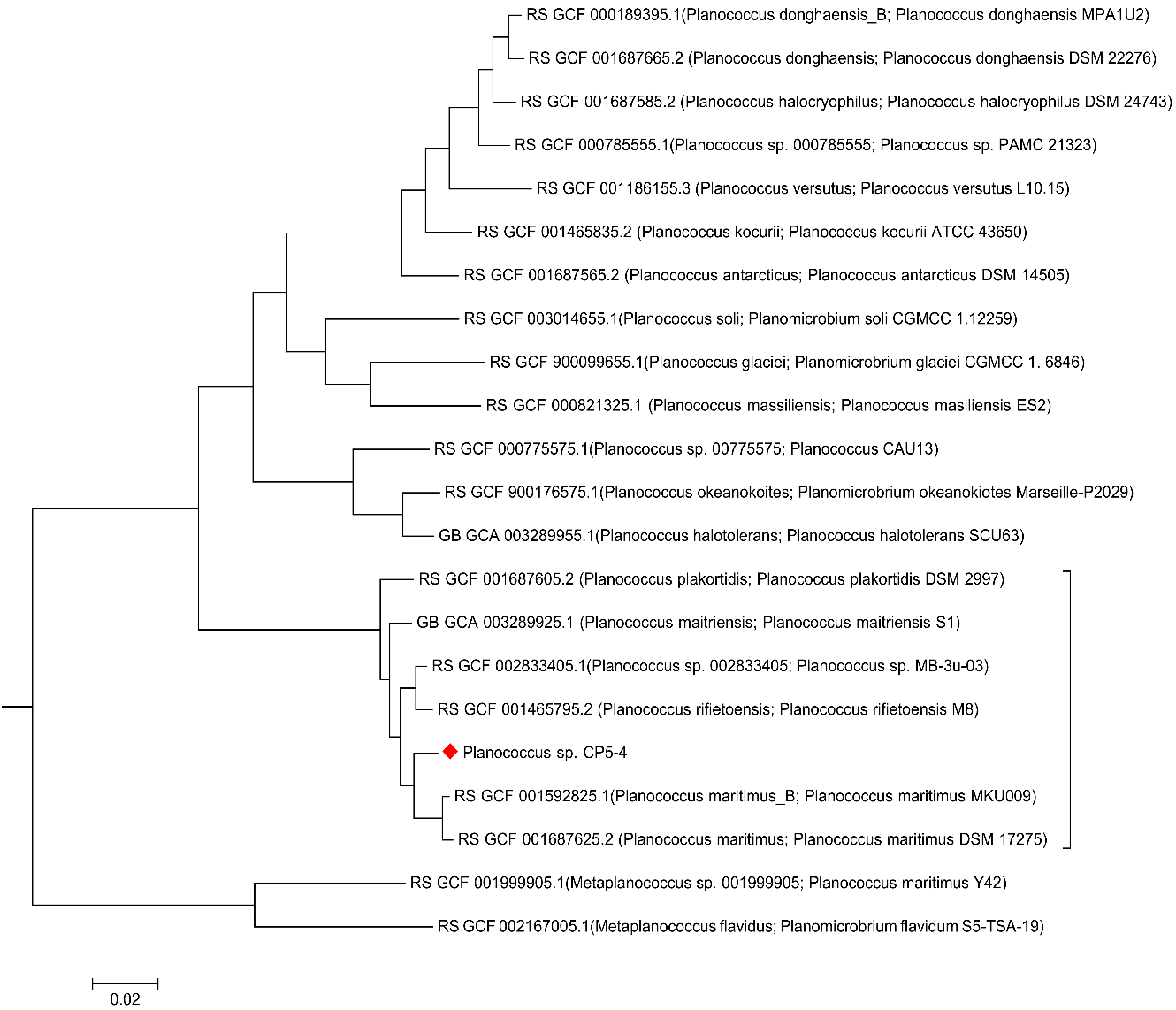


**Figure S1 Placement of the *Planococcus* CP5-4 strain in the GTDB bac 120 tree relative to other *Planococcus* species.** The genome identifiers of the organisms are shown on the tree branches, where appropriate, both the GTDB (release.95) and unfiltered NCBI taxonomy names are given in parenthesis. The tree is based on the topology of the genomes and not on bootstrap resampling as this is computationally prohibitive and consequently may over-classify genomes relative to manual curation based on unsupported affiliations of user genomes to reference taxa. Part of the GTDB bac120 genome tree is shown.

Table S4 Average nucleotide identity comparison between CP5-4 and closely related GTDB *Planococcus* species

| GTDB related reference | NCBI organism name | Genome id | ANI (%)^a^ | AF^b^ | Isolate Attributes | Geographical location and environment of origin | Reference |
| --- | --- | --- | --- | --- | --- | --- | --- |
| *Planococcus* sp*.* 002833405 | *Planococcus* sp. MB-3u-03 | GCF_002833405.1 | 89.49 | 0.83 | Gram-positive, orange/yellow, aerobic, non-sporulating, psychrotrophic coccoid bacterium. | Isolated from cold surface coastal water of the Pacific Ocean near Santa Cruz, CA (+37°0′12.32″N, −122°11′9.51″W) | (21) |
| *Planococcus* sp*.* CP5-4 | ND**^c^** | ND**^c^** | ND**^c^** | ND**^c^** | Gram-positive, orange, mesophilic, coccoid bacterium. | Isolated from brine samples from Cerebos crystallizer salt ponds in Velddrif, Western Cape, South Africa (S 32°47′10,632, E 18°10′9,499). | This study |
| *Planococcus rifietoensis* | *Planococcus rifietoensis* strain M8 | GCF_001465795.2 | 89.44 | 0.87 | Gram-positive, brilliant orange, mesophilic, aerobic/microaerophilic, non-sporulating coccoid bacterium. | Isolated from an algal mat collected from a sulfurous spring in Campania (Italy) (41.22852” N, 15.179250000000025 E”). | (82) |
| *Planococcus maitriensis* | *Planococcus maitriensis* strain S1 | GCA_003289925.1 | 87.57 | 0.82 | Gram-positive, orange, aerobic, psychrophilic, motile, non-sporulating, coccoid bacterium. | Isolated from a cyanobacterial mat sample collected from Schirmacher Oasis in Antarctica. | (18) |
| *Planococcus plakortidis* | *Planococcus plakortidis* DSM 23997 | GCF_001687605.2 | 86.80 | 0.82 | Gram-positive, yellow-orange, aerobic, non-spore-forming, mesophilic, motile, coccoid bacterium. | Isolated from a marine sponge [*Plakortis simplex* (Schulze)], collected at a depth of 30 m in the Bay of Bengal, off the coast of Gopalpur in the Indian state of Orissa (84° 43.907′ N and 18° 57.169′ E). | (83) |
| *Planococcus maritimus* | *Planococcus maritimus* DSM 17275 | GCF_001687625.2 | 86.54 | 0.83 | Gram-positive, strictly aerobic, motile, yellow-orange, psychrotrophic, coccoid bacterium. | Isolated from sea water of a tidal flat in Korea | (84) |
| *Planococcus maritimus_B* | *Planococcus maritimus* MKU009 | GCF_001592825.1 | 86.29 | 0.84 | Gram-positive, yellow, coccoid bacterium. | Isolated from surface marine waters of Pichavaram (11.45N, 79.79E), South East Coast, Tamil Nadu, India | (12) |

**^a^** GTDB determined percentage average nucleotide identity to *Planococcus*_B sp. CP5-4 query genome

**^b^** GTDB determined genome alignment fraction values for the query genome and the closet relative’s genome

**^c^** Not determined for the query genome.

Table S5 Genome property comparison between CP5-4 and related *Planococcus* species

| GTDB related reference | NCBI organism name | Genome id | Genome Size (bp) | G+C Content % | Total number of genes encoded | tRNAs |
| --- | --- | --- | --- | --- | --- | --- |
| *Planococcus* sp*.* 002833405 | *Planococcus* sp. MB-3u-03 | GCF_002833405.1 | 3 614 431 | 47.95 | 3 621 | 71 |
| NA^a^ | *Planococcus* sp. CP5-4 | NA**^a^** | 3 488 448 | 47.50 | 3 557 | 59 |
| *Planococcus rifietoensis* | *Planococcus rifietoensis* strain M8 | GCF_001465795.2 | 3 505 011 | 48.45 | 3 507 | 72 |
| *Planococcus maitriensis* | *Planococcus maitriensis* strain S1 | GCA_003289925.1 | 3 145 619 | 49.91 | 3 148 | 50 |
| *Planococcus plakortidis* | *Planococcus plakortidis* DSM 23997 | GCF_001687605.2 | 3 261 818 | 49.98 | 3 241 | 70 |
| *Planococcus maritimus* | *Planococcus maritimus* DSM 17275 | GCF_001687625.2 | 3 280 721 | 47.17 | 3 241 | 71 |
| *Planococcus maritimus_B* | *Planococcus maritimus* MKU009 | GCF_001592825.1 | 3 251 644 | 47.27 | 3 259 | 64 |

# ^a^ Not available

# Random mutagenesis and identification of carotenoid biosynthetic genes


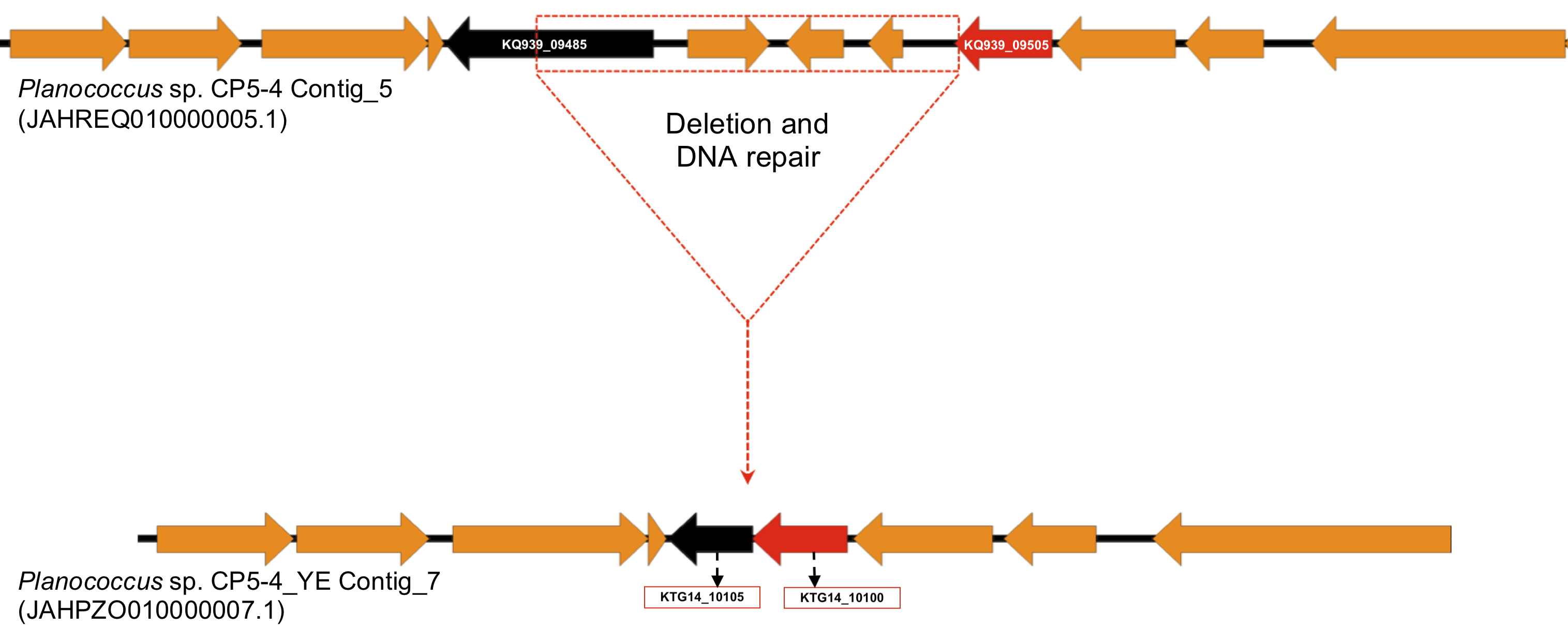


**Figure S2 Deleted 3 Kb sequence region from the wild type CP5-4 strain's genome to produce a truncation of *crt*P and expression of the yellow mutant phenotype.** The black coloured ORF represents *crt*P while the red ORF represents the flavodoxin reductase gene. The respective locus tags for the ORFs are also shown. Figure not drawn to scale.


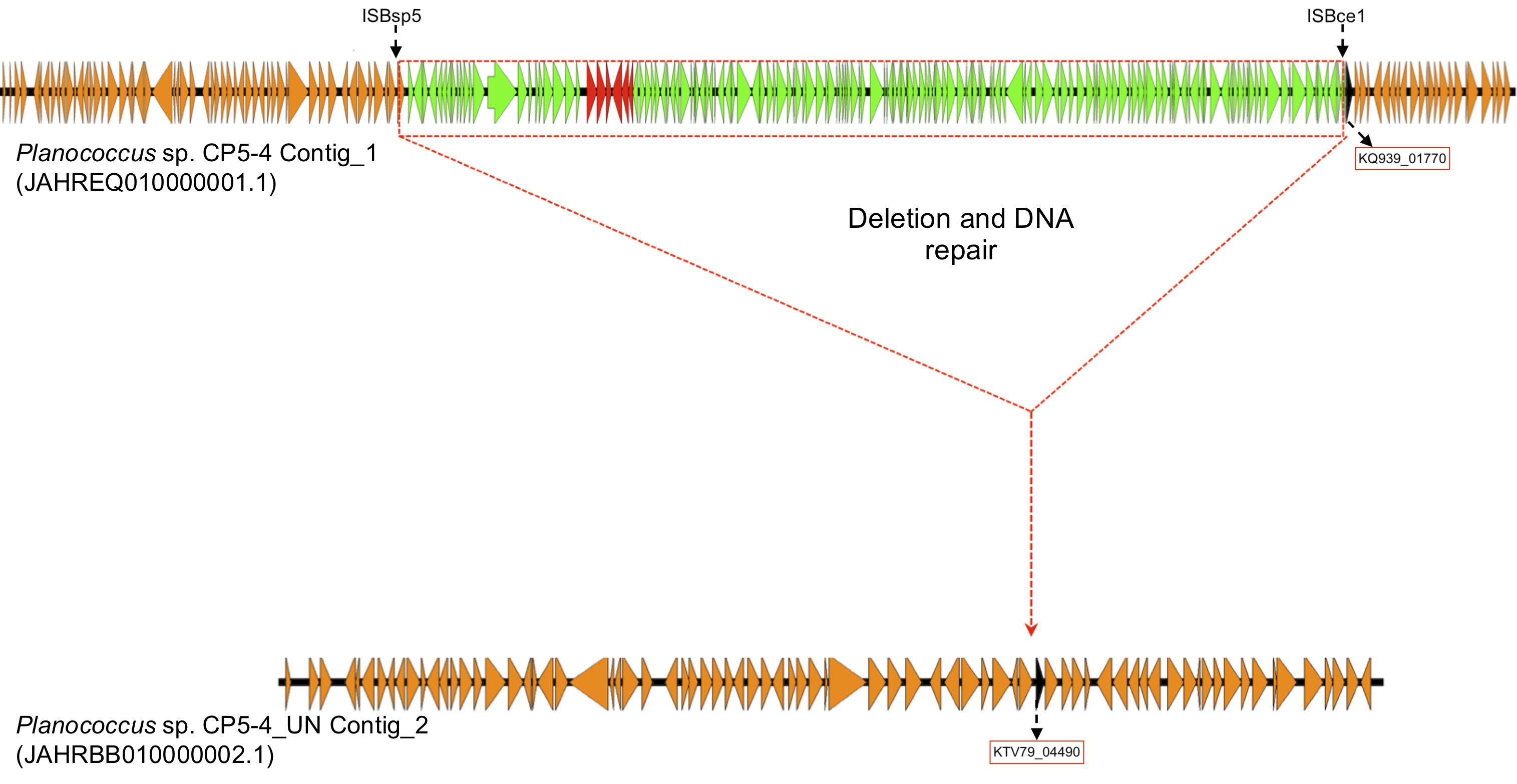


**Figure S3 Deleted 146.691 Kb sequence region from the wild type CP5-4 strain's genome to produce the unpigmented mutant phenotype.** The red ORFs represent the predicted carotenoid biosynthetic gene cluster while the green ORFs represent the additional genes that were also deleted as a result of DNA repair following MMS mutagenesis. The position of two insertion sequences ISBsp5 and ISBce1 (Table S4) that may have mediated the deletion are shown. Figure not drawn to scale.

Until recently, SNPs were thought to be the predominant form of genomic variation induced by exposure to DNA alkylating agents such as MMS (79). However, recent developments and applications of genome wide technologies have led to the discovery of thousands of copy number variants (CNVs; classified as SVs) in genomes (48).

Copy number variants are defined as a duplication or deletion (i.e., a gain or loss of a genomic DNA segment relative to a reference sample) measuring greater than 1 Kb in size (80). According to (48), copy number variant hotspots exist in microbial genomes where they mediate the frequent rearrangement of these genomes leading to a discontinuity in the DNA of strains of the same species. Thus, enabling the development of new phenotypes in response to environmental changes. Additionally, mobile genetic elements are frequently found near these hotspots, suggesting a mechanism for generating genomic instability following activation (48). Chemical mutagens such as MMS that cause little chromosome breakage but add bulky adducts to the DNA can induce the SOS response, which leads to the activation, and the transposition of the mobile genetic elements. Consequently, recombination following the generation of 2 copies of the same sequence in the same orientation during transposition can result in the deletion of the DNA sequence between them (48). This process may have mediated the deletion of the 146691 bp fragment from the genome to give the unpigmented phenotype after MMS mutagenesis.

## Genes involved in unsaturated odd chain fatty acid biosynthesis in CP5-4

Table S6 Locus tags in CP5-4 involved in unsaturated fatty acid biosynthesis

| Locus tag | Gene product | Function |
| --- | --- | --- |
| KQ939_06015 | Methylmalonyl-CoA mutase large subunit MutB (EC 5.4.99.2). | Catalyzes the reversible isomerisation of methylmalonyl‐CoA to succinyl‐CoA, which is later converted to propionyl-CoA for the biosynthesis of unsaturated fatty acids in bacteria. |
| KQ939_06020 | Methylmalonyl-CoA mutase small subunit, MutA (EC 5.4.99.2). |  |
| KQ939_06010 | YgfD; sleeping beauty mutase | Protein that forms a complex with methylmalonyl-CoA mutase in a pathway for conversion of succinyl-CoA to propionyl-CoA |
| KQ939_01020 | FabZ; 3-hydroxyacyl-[acyl-carrier-protein]-dehydratase | Catalyzes the dehydration of short chain beta-hydroxyacyl-ACPs and long chain saturated and unsaturated beta-hydroxyacyl-ACPs. |
